# Integrating Standard Precipitation Index and Normalised Difference Vegetation Index for near-real-time drought monitoring in Eswatini

**DOI:** 10.4102/jamba.v11i1.917

**Published:** 2019-12-12

**Authors:** Daniel H. Mlenga, Andries J. Jordaan, Brian Mandebvu

**Affiliations:** 1 Disaster Management Training and Education Centre for Africa, Faculty of Natural and Agricultural Sciences, University of the Free State, Bloemfontein, South Africa; 2 Institute of Development Studies, National University of Science and Technology, Bulawayo, Zimbabwe

**Keywords:** drought, Standard Precipitation Index, Normalised Difference Vegetation Index, drought monitoring, early warning

## Abstract

Eswatini, as the rest of southern Africa, is being frequented by drought over the last decade, and modelling experts are predicting that drought years will become more and severe. The expected increase in extreme climatic events makes the use of drought indices essential for drought monitoring and early warning. To enable Eswatini to better prepare, analyse and respond to drought, this study analysed the use of Normalised Difference Vegetation Index (NDVI) and Standard Precipitation Index (SPI) for near-real-time drought monitoring through the development of a model for drought severity. Meteorological stations across all agro-ecological zones with data for the period 1986–2017 were selected for analysis. The SPI computation was achieved through DrinC software. Primary NDVI data sources were CHIRPS gridded rainfall dataset and the MODIS NDVI CMG data. Results of the 3-month SPI indicated that moderate droughts were experienced in 1990/1991, 2005/2006, 2011/2012, 2012/2013 and 2015/2016. The Highveld and Middleveld had the lowest drought occurrence percentage of 3.3%, whereas the likelihood of having a moderate, severe and extreme drought was higher in the Lowveld. The study determined a positive correlation between the SPI and the NDVI at 3-month time scale, and a value of *Y* (drought severity) greater than 0.54 indicated a significant dry spell and could be used as a drought trigger threshold for early warning. The combined use of NDVI and SPI was deemed capable of providing a near-real-time indicator for drought conditions allowing planners to provide timely information for drought preparedness, mitigation and response planning, thereby helping to lower the eventual drought relief costs, protect food security and reduce the humanitarian impact on the population.

## Introduction

Eswatini, as the rest of southern Africa, is being frequented by drought over the last decade. This phenomenon has become more frequent and severe leading to the increased interest by climate scientists as well national governments and UN agencies as devastating effects of a drought event on the local economy and life of the people can be severe and needs strategic and emergency planning and response. Climate scientists and modelling experts have predicted that drought years will become more and severe in southern Africa (Carty 2017), and this has already been experienced with the droughts in 2001, 2005, 2007 and 2015 in Eswatini.

The impact of drought in Eswatini can be severe only because almost 70% of the population rely on rain-fed agriculture (SNVAC 2015) and over 40% of the country fall in the Lowveld agro-ecological zone which receives an annual average rainfall of below 500 mm. The Eswatini Vulnerability Assessment reports have indicated that yields, especially in the Lowveld, can be reduced by over 50% during drought years, thereby affecting the overall country food security situation. This reduction in agriculture production, especially for the maize (the staple crop), results in the government importing grain and the UN and non-governmental organisations providing 20% – 30% of the population with food aid. The extent of the food aid distributed as well as imports needed requires effective and timely planning by the government and stakeholders, ensuring that the required food is available on time and the populace does not go hungry. This makes the aspect of drought monitoring and early warning critical.

Accurately and efficiently monitoring drought has been difficult mainly because of the difficulty in determining its onset, development and end. The expected increase in extreme climatic events, especially drought, makes the use of drought indices essential for drought monitoring. Integrating retrospective analysis with real-time monitoring could be extremely beneficial in the development of response, mitigation strategies and awareness plans. Traditionally drought disaster management took a “crisis management” approach, where focus was on actions taken immediately before, during and shortly after a disaster (WMO 2006). Embracing of the Hyogo Framework for Action 2005–2015 (UNISDR 2005) globally has led to a fundamental change in drought disaster risk management from emergency response to an inclusive approach including preparedness, where drought monitoring is important, and preventive approaches to acknowledge and reduce risk (WMO 2014).

### Overview of drought monitoring and early warning systems

The capacity to monitor and predict the drought attributes (onset, frequency and severity) is fundamental for spatial–temporal (drought) analysis. Accurate monitoring of the spatial and temporal distribution of the onset, extension and severity of drought is an essential instrument for informed and calculated decision-making in drought mitigation and management (Covele & Sannier 2005). Most commonly, drought indices are used to monitor drought conditions with many indices in existence and utilised to forecast the possible development and progression of an existing drought. There are several indices that measure how much precipitation for a given period of time has deviated from historically established norms. Although none of the major indices are inherently superior to the rest in all circumstances, some indices are better suited than others for certain uses (NDMC 2014). Drought indices mostly are functions of precipitation and/or temperature, river discharge (Zehtabian et al. 2013). Precipitation is the most commonly used indicator for drought monitoring.

Well-established drought indicators exist that include the Percent of Normal, Palmer Drought Severity Index (PDSI) (Palmer 1965), the Standard Precipitation Index (SPI), the Standardised Precipitation Evaporation Index (SPEI) (McKee, Doesken & Kleist 1993), Deciles (Gibbs 1967), Crop Moisture Index (CMI) (Palmer 1968) and Reconnaissance Drought Index (RDI) (Tsakiris & Vangelis 2005). The SPI is one of the most common indexes used to monitor drought because it presents a quick measure with minimal data requirements. A single numeric value is assigned to precipitation that can be compared across regions and different climates (Komuscu 1999; MacKee et al. 1993).

Reliance, however, on weather data alone might not be adequate to monitor drought in all the areas, especially in southern Africa, where data can be incomplete, unavailable, untimely and unreliable. It is therefore necessary to complement weather-based data with satellite imagery to identify the spatial and temporal dimensions of drought, and to attain a complete, up-to-date, comprehensive coverage of drought conditions (Peters et al. 2002). Satellite images can be utilised for determining the spatial and temporal variability of drought hazard, and the vulnerability of water resources, vegetation systems and society to drought is essential for any early warning and drought monitoring tool. The use of satellite technology is therefore significant for drought information systems for Africa so as to generate seasonal or monthly drought hazard maps; drought vulnerability maps; real-time drought monitoring based on indicators and real-time drought early warning.

Utilisation of different satellite-based drought monitoring indices such as Normalised Difference Vegetation Index (NDVI) and its derivatives such as Vegetation Condition Index (VCI), Standard Vegetation Index (SVI), Vegetation Productivity Index (VPI) and other indices such as the FAO Agriculture Stress Index (ASI) are essential for drought monitoring or the detection of near-real-time onset, evolution, intensity and duration of drought in Eswatini and southern Africa. In other countries, the use of remote sensing for drought monitoring is one of the many examples where derived satellite data are currently used to derive police and government planning decisions. The NDVI, the Water Supply Vegetation Index (WSVI), VCI and SVI have been used for vegetation monitoring, crop yield assessment and estimation, early warning systems and drought monitoring (Bhuiyan 2004; Brown et al. 2008; Covele et al. 2005; Jain et al. 2010; Moulin Bondeau & Delecolle 1998; Sing et al. 2003). The study focussed on the integration of NDVI and SPI for near-real-time drought monitoring in Eswatini.

### Drought events in Eswatini

Drought is a normal part of southern Africa’s climate, and it is highly unusual for drought not to occur somewhere in southern Africa each year (Uganai 1994). During the last century, southern Africa, and Eswatini in particular, has been characterised by an increased frequency of droughts (EM-DAT 2018). Recorded drought years in the southern African region include that of 1982–1983, 1987–1988, 1991–1992, 1994–1995, 1997–1998, 2002–2003 (Covele & Sannier 2005), 2005–2006, 2007–2008, 2009–2010, 2012–2013, 2015–2016 (EM-DAT 2018). Drought in Eswatini has almost followed a similar pattern to the whole of southern Africa. According to drought disaster declarations made by the government, and extreme drought events recorded for 1900–2016 in EM-DAT, the drought years were experienced in Eswatini in the years 1981, 1984, 1990, 2001, 2007 and 2016. Droughts have impacted the country differently in space and time (SNVAC 2004, 2006, 2007, 2008, 2016). The drought in 1983 had the largest human loss of 500 people, whereas the 2005/2006 drought affected 410 000 people. With a population estimated at 1 403 362, it indicates 13% of the population being affected, which is significant considering that Eswatini is affected by many other hazards, most significantly HIV/AIDS (CIA 2018).

### Standard Precipitation Index

McKee developed the SPI during the early 1990 (McKee et al. 1993). The WMO in 2009 recommended SPI as the main meteorological drought index that countries should use to monitor and follow drought conditions (Hayes et al. 2011). The index is recommended because it allows the comparison between different climates and locations. It can be used to analyse drought or anomalously wet periods at a particular timescale for any location in the world with daily precipitation records (McKee, Doesken & Kleist 1995; Moreira et al. 2008). These timescales, days, weeks, months and years reflect the impact of drought on the availability of the different water resources. The index can identify various drought types: hydrological, agricultural or environmental. Using the SPI as indicator for drought monitoring, early warning drought disaster declaration will limit the arbitrary decision-making of politicians with scientifically based criteria.

### The Normalised Difference Vegetation Index

Normalised Difference Vegetation Index is a remote sensing-based index that measures vegetation conditions (Rouse et al. 1974). It uses the advanced very high-resolution radiometer (AVHRR) reflected red and near-infrared channels to calculate if the vegetation is healthy, or unhealthy and sparse (e.g. suffering from drought or insect infestation) (Zargar et al. 2011). The NDVI is generally recognised as a good indicator of terrestrial vegetation productivity and, for a long time, has been used for estimation of net primary production, crop growth conditions, land cover, crop yield estimation, rainfall and drought monitoring and early warning systems (Singh et al. 2003). It is also effective in monitoring climate variability, land use and vegetation type (Covele & Sannier 2005). The identification of areas with vegetation more sensitive to drought can be applicable in drought risk management (Alamdarloo Manesh & Khosravi 2018), and be used to map areas that are affected by drought.

## Research methods and design

### Study area

**FIGURE 1:**
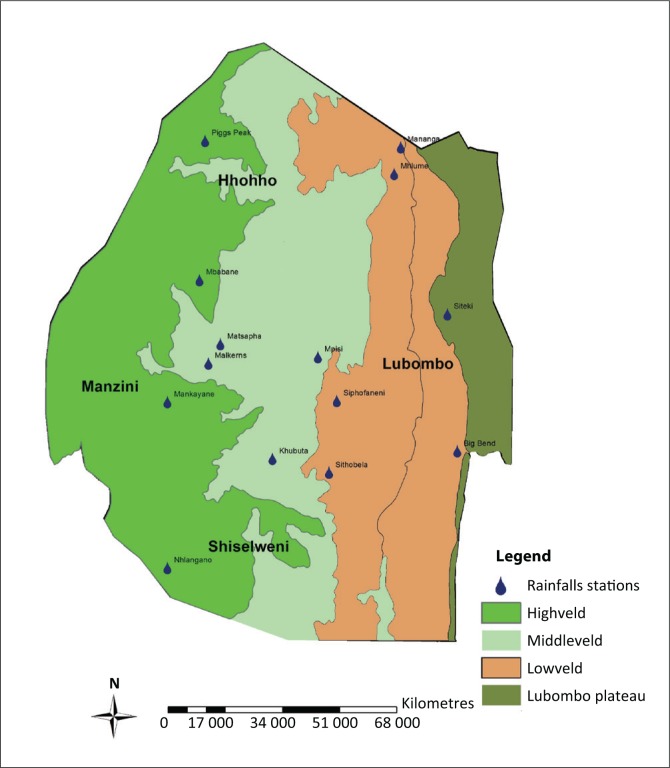
Map of Eswatini with agro-ecological zonation and the rainfall stations.

The country is classified into four agro-ecological zones (AEZ) – *Highveld, Middleveld, Lowveld* and *Lubombo Range* – based on elevation, landforms, geology, soils, climate and vegetation. The rainy season is from mid-October to mid-April, with mean annual rainfall ranging from 700 mm to 1500 mm in Highveld to 200 mm in the southern Lowveld (Table 1). These large ranges indicate the fluctuating nature of Eswatini’s climate and make the country very vulnerable to meteorological hazards such as drought and floods (Figure 1).

**TABLE 1:** Rainfall in ecological zones of Eswatini.

Agro-ecological zone	Average rainfall
Highveld	700–1550
Middleveld	550–850
Lowveld	200–550
Lubombo Plateau	550–850

*Source*: FAO AQUASTAT Survey, 2005, *Irrigation in Africa in figures*, viewed 04 March 2017, from http://www.fao.org/ag/aquastat.

## Applied methodology

### Computation of Standard Precipitation Index

The SPI was used for drought monitoring for the time series from the period 1986 to 2017. Representative meteorological stations of the Eswatini Meteorological Service were selected, with good data. The stations covered all agro-ecological zones and administrative regions in Eswatini. Only stations with full data were considered for analysis. There was therefore no data filling or corrective homogeneity enforced. The SPI computation was achieved through the use of DrinC software, and drought classification (Table 2) was based on the SPI classification (McKee et al. 1993) where a drought event occurs any time. The SPI is continuously negative and reaches an intensity of −1.0 or less (McKee et al. 1993, 1995).

**TABLE 2:** Drought classification based on Standard Precipitation Index.

SPI values	Class
≥ 2	Extremely wet
1.5–1.99	Very wet
1.0–1.49	Moderately wet
−0.99 to 0.99	Near normal
−1 to −1.49	Moderately dry
−1.5 to −1.99	Very dry
≤ 2	Extremely dry

*Source*: McKee, T.B., Doesken, N.J. & Kleist, J., 1993, ‘The relationship of drought frequency and duration to time scales’, in *Proceedings of the 8th Conference on Applied Climatology*, American Meteorological Society, Boston, MA, January, Vol. 17, No. 22, pp. 179–183.

SPI, Standard Precipitation Index.

The selection of software was based on its simplicity, such that it can be easily adopted for the use in Eswatini. DrinC is a user-friendly tool software package which was developed for providing a simple, though adaptable, interface for the calculation of several drought indices (Tigkas, Vangelis & Tsakiris 2015). The software operates on Windows platform and is programmed in Visual Basic. A series of data, at least for a period of 30 years, was used to determine the 3-month (October, November, March) SPI values.

#### Normalised Difference Vegetation Index application and mapping methodology

The primary data sources were CHIRPS gridded rainfall dataset produced by the Climate Hazards Group at the University of California, Santa Barbara, and the MODIS NDVI CMG data made available by NOAA-NASA and the NDVI based on Global Agricultural Monitoring (GLAM). The NDVI data in use are from the MODIS platforms Terra and Aqua, which provide global coverage since 2000 (Terra) and mid-2002 (Aqua) at about 5-km resolution with a temporal frequency of overlapping 16-day periods. Normalised Difference Vegetation Index is based on GLAM, a collaboration between the United States Department of Agriculture (USDA) and the National Aeronautics and Space Administration (NASA), University of Maryland, Department of Geography, Goddard Space Flight Centre (GSFC) and the USDA Foreign Agricultural Service (FAS), that used satellite data and data products to monitor agriculture worldwide and to locate and keep track of natural hazards such as short- and long-term droughts, floods and persistent snow cover which impair agricultural productivity. For effective analysis and presentation, monitoring was done on a dekadal basis, with three dekads of January during the drought years.

### Ethical considerations

This article followed all ethical standards for research without direct contact with human or animal subjects.

## Results and discussion

### Drought severity spatio-temporal dynamics based on Standard Precipitation Index

**FIGURE 2:**
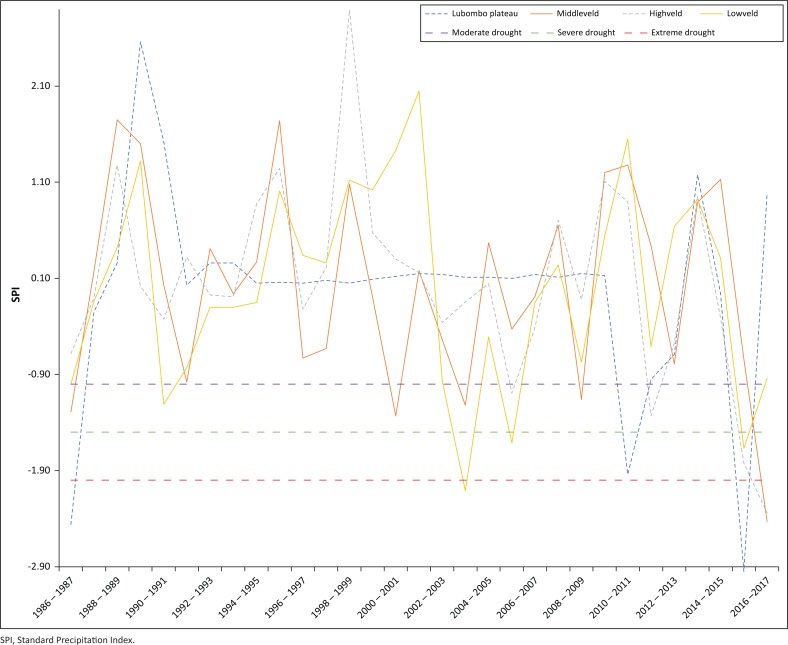
Three-month Standard Precipitation Index values for the Highveld, Middleveld, Lowveld and Plateau agro-ecological zone.

The 3-month SPI results indicate that moderate droughts were experienced in 1990/1991, 2005/2006, 2011/2012, 2012/2013 and 2015/2016 rainfall seasons. Comparing the 3-month SPI across AEZs, most drought events were experienced in the Middleveld and Lowveld zones. Moderate droughts were experienced in 1990/1991, 2005/2006, 2011/2012, 2012/2013 and 2015/2016 rainfall seasons. Eswatini suffered a severe drought in the 2015 and 2016 season, which was consistent with declaration of drought emergencies in the southern African region, largely because of the El Niño, one of the strongest on record (WFP 2016). When the 3-month SPI was calculated for the different AEZs, there were parallels with the drought periods that were declared and documented in the EM-DAT database. The spatial and temporal differences for moderate drought typify most of the droughts that were declared and experienced in the country (Figure 2).

### Drought severity temporal and spatial dynamics based on Normalised Difference Vegetation Index

**FIGURE 3:**
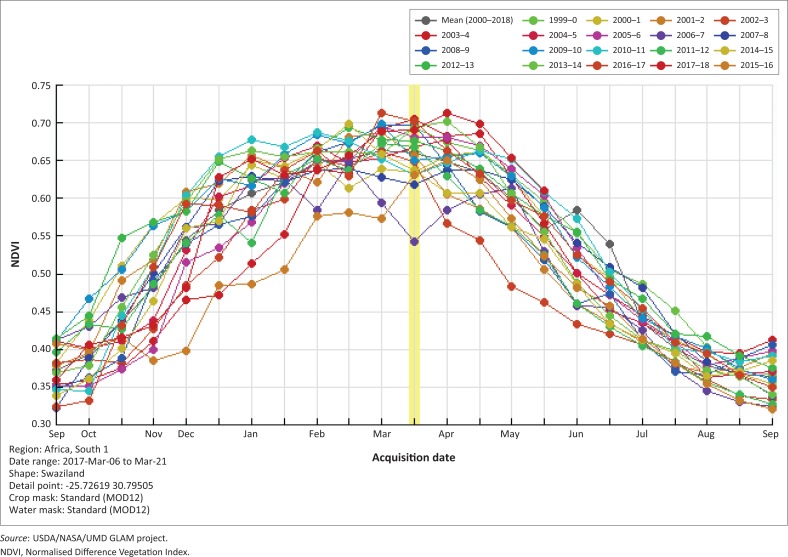
MODIS NDVI (Terra) (MOD44 16 days) graph for 2000–2018.

**FIGURE 4:**
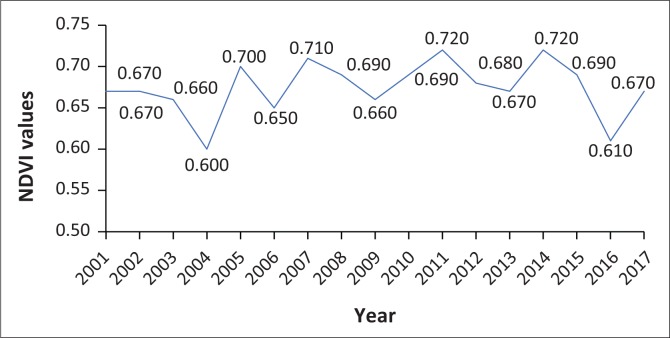
Lowest MODIS NDVI (Terra) (MOD44 16 days) for the month of January for 2000–2018.

The MODIS NDVI average values for Eswatini indicate that for the months of January–May the NDVI values are high, indicating the growing season. The peak vegetative period is from February to April (GOS 2016), where the NDVI values fall between 0.65 and 0.75 (Figure 3), which correspond to dense vegetation. Analysing the month under study, January, it is observed that low-NDVI values correspond to the drought years (Figure 4) that have been indicated for Eswatini.

The 2015–2016 season experienced low rainfall which is reflected by the year 2016 having the lowest NDVI. This is further corroborated by the declaration of a drought as national emergency in 2016 by the Eswatini government.

### Relationship between Normalised Difference Vegetation Index and Standard Precipitation Index

**FIGURE 5:**
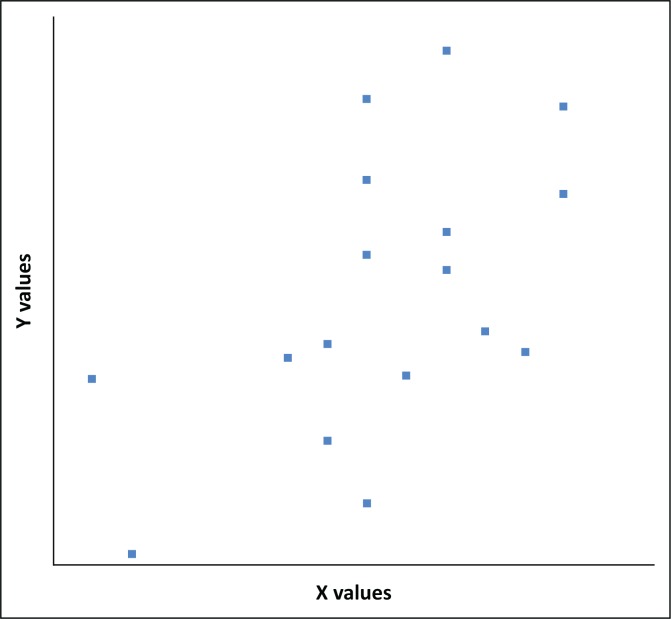
Relationship between Normalised Difference Vegetation Index and Standard Precipitation Index

The Pearson product-moment correlation coefficient (or Pearson correlation coefficient) was used to analyse the relationship between NDVI and SPI to see if the indices can be used in a model to determine drought severity. Using the formula below for calculating the correlation coefficient, the *r* value was calculated for the months of December for SPI values and NDVI for January for the selected drought years (Table 3; Figure 5). The scatter points are close to the line, indicating that the two variables have a positive correlation, which indicates only a moderate to positive linear relationship between the variables.

$$\sigma_{XY}=\frac{\sum_{i=1}^{n}\left(X_{i}-\bar{X})(Y_{i}-\bar{Y}\right)}{\sqrt{\sum_{i=1}^{n}{\left(X_{i}-\bar{X}\right)}^{2}\sum_{i=1}^{n}{\left(Y_{i}-\bar{Y}\right)}^{2}}}$$

Result details and calculation *X* values:

***X* Values**

$$\begin{array}{l} \sum =11.46\\ \bar{X}=0.674\\ \sum_{i=1}^{n}{\left(X_{i}-\bar{X}\right)}^{2}=SS_{x}=0.018 \end{array}$$

**Y Values**

$$\begin{array}{l} \sum =-0.41\\ \bar{Y}=-0.024\\ \sum_{i=1}^{n}{\left(Y_{i}-\bar{Y}\right)}^{2}=SS_{y}=16.938 \end{array}$$

**X and Y Combined**

$$\begin{array}{l} N=17\\ \sum_{i=1}^{n}(X_{i}-\bar{X})(Y_{i}-\bar{Y})=0.303 \end{array}$$

**R Calculation**

$$\begin{array}{l} \sigma_{XY}=\frac{\sum_{i=1}^{n}\left(X_{i}-\bar{X})(Y_{i}-\bar{Y}\right)}{\sqrt{SS_{x}SS_{y}}}\\ \sigma_{XY}=\frac{0.303}{\sqrt{\left(\left(0.018)(16.938\right)\right)}}=0.5544 \end{array}$$

**TABLE 3:** Normalised Difference Vegetation Index and Standard Precipitation Index for selected drought years.

Year	NDVI (January)	SPI-3 (December)
2017	0.67	−1.54
2016	0.61	−1.90
2015	0.69	0.16
2013	0.67	0.27
2011	0.72	1.35
2008	0.69	0.45
2007	0.71	−0.44
2006	0.65	−0.48
2005	0.70	−0.28

NDVI, Normalised Difference Vegetation Index; SPI, Standard Precipitation Index.

The value of *R* was 0.5544 which showed also a positive correlation, which meant that high *X* variable scores go with high *Y* variable scores (and vice versa). The value of *R*^2^, the coefficient of determination, was 0.52 (Table 4). By calculating the correlation between SPI and NDVI, it can be clearly noticed that they show a positive correlation at 3-month time scale. The results are consistent with results reported by Ji and Peters (2003), Dutta, Kundu and Patel (2013), Tamassoki et al. (2014), Dodamani, Anoop and Mahajan (2015), Yang et al. (2015) and Khosravi et al. (2017), who all confirmed that highly significant correlations were obtained between current NDVI and SPI of various time lags at the significant level of 95%.

**TABLE 4a:** Summary of outputs.

Regression Statistics	Value
Multiple *R*	0.52
*R* square	0.28
Adjusted *R* Square	0.12
Standard error	0.47
Observations	18

**TABLE 4b:** Summary of outputs – ANOVA.

Model	df	SS	MS	F	Significance F
Regression	3	1.18	0.39	1.78	0.20
Residual	14	3.10	0.22	-	-
Total	17	4.28	-	-	-

**TABLE 4c:** Summary of outputs.

Variables	Coefficients	Standard error	*t* stat	*p*	Lower 95%	Upper 95%
Intercept	5.28	8.24	0.64	0.53	−12.39	22.96
NDVI	9.63	4.23	2.28	0.04	0.56	18.71
Temperature	-0.48	0.38	−1.25	0.23	−1.30	0.34
SPI-3	−0.17	0.12	−1.47	0.16	−0.42	0.08

NDVI, Normalised Difference Vegetation Index.

### Near-real-time drought monitoring

The analysis of remote sensing-based drought indices and SPI can provide a far-reaching understanding of the spatio-temporal dynamics of large-scale drought patterns. Because of the strong positive correlation between NDVI and SPI, the two indices can be used to monitor and detect drought, thereby providing early warning information to stakeholders. To quantify the strength and duration of droughts that could have a significant impact on the population and the economy, the study derived statistical threshold based on parameters from NDVI, time series of SPI-3. Normalised Difference Vegetation Index and 3-month (October–December) SPI, and precipitation data from 2001 and 2017 were used to develop the statistical threshold to classify drought periods. Normalised Difference Vegetation Index for all the drought periods, for the month of January, averaged 0.66. The 3-month SPI values for the month of December ranged from 1.76 to −1.90.

The available data were used to develop as a drought trigger threshold for early warning. The method of analysis was the least squares (LS), which is simply a minimisation of the sum of the squares of the deviations of the observed response from the fitted response (Naoum & Tsanis 2003). This involved the initial assumption that a certain type of relationship, linear in unknown parameters, holds. With drought severity (*Y*) being the dependent (response) variable, the model function is of a specified form that involves both the predictor variables (NDVI and SPI) and the parameters. Interaction effects between the variables can also be considered. The unknown parameters or thresholds were estimated with assumptions with the help of available data so that a fitted equation was obtained. In the model, drought determination was based on three main parameters, SPI, NDVI and rainfall.

The general form of the final model was

$$Y=\beta_{0}+\beta_{1}X_{1}+\beta_{2}X_{2}+\beta_{3}X_{3}$$

where *Y* is drought severity, *X*_1_ is NDVI, *X*_2_ is SPI and *X*_3_ is temperature (ºC).

Based on the model, the study determined that the value of *Y* (drought severity) that is greater than 0.54 indicates a significant dry spell, meaning the value will be recommended to be used as a drought trigger threshold for early warning. High *Y* values for the years 2007 and 2016 coincide with the two strongest El Niño events and one remarkable La Niña episode in 2010 and 2011 rainfall season. Similarly, the retrospective analysis of agriculturally relevant droughts over Africa shows that major drought events, which are mentioned in the literature or registered in the EM-DAT disaster database of 2018, are largely mirrored in the data in Table 5 which indicate the *Y* values for all the years under study.

**TABLE 5:** Drought trigger threshold determination.

Year	Drought severity (*Y*)	Drought declaration status†	Recognised droughts based on yield and vulnerability†
2016–2017	0.356157	-	-
2016–2017	0.06165	-	-
2015–2016	0.538125	Official declaration	√
2014–2015	0.635436	-	√
2013–2014	0.239596	-	-
2012–2013	0.34424	-	-
2011–2012	0.599801	-	√
2010–2011	0.22795	-	-
2009–2010	0.546404	-	-
2008–2009	0.651257	-	√
2007–2008	0.690508	Official declaration	√
2006–2007	0.295794	-	-
2005–2006	0.673756	Official declaration	√
2004–2005	0.027779	-	-
2003–2004	0.272383	-	-
2002–2003	0.365377	-	-
2001–2002	0.385711	-	-

*Source:* Adapted from EM-DAT, 2018, *The OFDA/CRED International Disaster Database*, viewed 04 March 2018, from www.em-dat.net; Swaziland National Vulnerability Assessment Committee (SNVAC), 2004, *Swaziland national vulnerability assessment*, Mbabane; Swaziland National Vulnerability Assessment Committee (SNVAC), 2007, *Swaziland national vulnerability assessment*, Mbabane; Swaziland National Vulnerability Assessment Committee (SNVAC), 2016, *Swaziland national vulnerability assessment*, Mbabane.

† , Data obtained from table reference sources.

## Conclusion

Eswatini is being frequented by drought over the last decade. Accurate monitoring of the spatial and temporal distribution of the onset, extension and severity of drought is an essential instrument for informed and calculated decision-making. This study analysed the use of NDVI and SPI for near-real-time drought monitoring in Eswatini. Similar research by Hayes et al. (1999) found out that because of the SPI versatility, it can be calculated on any timescale, thereby giving it the ability to monitor drought conditions. Jain et al. (2010) also demonstrated that remote sensing can be used to relate drought conditions when correlated with precipitation-based drought indices. The capacity to monitor and predict the drought attributes (onset, frequency and severity) is fundamental for spatial-temporal (drought) monitoring. According to the results of this research, the combined use of NDVI and SPI was deemed capable of providing a near-real-time indicator for drought conditions within varying agro-ecological zones and time periods. Results of the 3-month SPI indicated that the Highveld and Middleveld had the lowest drought severity percentage and the likelihood of having a moderate, severe and extreme drought was higher in the Lowveld. The results are compatible with findings by Jain et al. (2010), who determined that drought affects nearly all climatic zones with semi-arid regions being especially susceptible to drought because of their low annual precipitation and sensitivity to climate variability. The frequency, severity and temporal of the drought events across the different agro-ecological zones make the aspect of drought monitoring and early warning critical for drought mitigation and management.

The positive correlation between the SPI and the NDVI enabled the use and optimisation of precipitation and remote sensing vegetation indices for analysing the spatial and temporal variability of drought and finding the positive and linear relationship between SPI and NDVI. Ji and Peters (2003), Dutta et al. (2013) and Khosravi et al. (2017) in their research also confirmed the positive relationship between the NDVI and SPI for drought monitoring; the more rainfall, the better quality of vegetation cover. The study developed trigger threshold, value of *Y* = 0.54 for drought severity (dry spell) obtained from the relationship between SPI for December and NDVI for January. Values of *Y* greater than 0.54 should therefore trigger drought disaster management stakeholders to initiate extensive drought mitigation planning including crop and vulnerability assessments to confirm if there is a drought occurring then what will be the likely impacts, before the negative impacts start to be felt by the population. The study lucidly shows that the use of SPI and NDVI, incorporated with the use of drought early warning trigger threshold, has a great potential in drought monitoring through early warning. This moves away from a crisis management to a proactive disaster risk reduction approach, allowing planners to provide very useful and timely information for drought preparedness, mitigation and response planning. Drought preparedness and risk mitigation will help lower the eventual drought relief costs, protect food security and reduce the humanitarian impact on the population.
